# Physical activity in depressed and non-depressed patients with obesity

**DOI:** 10.1007/s40519-016-0347-8

**Published:** 2017-02-10

**Authors:** Christian Sander, Patrick Ueck, Roland Mergl, Gemma Gordon, Ulrich Hegerl, Hubertus Himmerich

**Affiliations:** 1 Department of Psychiatry and Psychotherapy, University Hospital Leipzig, Leipzig, Germany; 2 Department of Psychological Medicine, King’s College London, 103 Denmark Hill, London, SE5 8AF UK; 3 Leipzig University Medical Center, IFB Adiposity Diseases, Leipzig, Germany

**Keywords:** Steps, Activity, Actigraphy, Depression, Obesity

## Abstract

**Purpose:**

Obesity and depression have both been shown to be associated with reduced physical activity (PA). However, most studies have not applied objective measures to determine PA in patients. Moreover, to our knowledge, no studies are available comparing depressed and non-depressed patients with regard to PA.

**Methods:**

We investigated PA in 47 patients with both obesity and depression, 70 non-depressed patients with obesity, and 71 non-depressed and non-obese healthy control participants using the SenseWear™ Armband (SWA) with walked steps per day and metabolic equivalents (MET) as parameters for PA.

**Results:**

Depressed as well as non-depressed patients with obesity showed a significantly reduced PA as reflected by walked steps as well as reduced METs. Healthy controls walked a mean of 11,586 ± 3731 (SD) steps per day, whereas non-depressed patients with obesity accumulated 7283 ± 3547 and patients with both obesity and depression recorded only 6177 ± 3291 steps per day. However, the difference between depressed and non-depressed patients with obesity did not reach statistical significance either in terms of walked steps or with regard to METs.

**Conclusions:**

Obesity seems to be associated with a substantial reduction of PA and energy expenditure, whereas the effect of an additional depressive disorder was comparably small. Even though depression did not have any statistically significant effect on steps and METs per day in this study with obese patients, it could be clinically relevant for an individual patient.

## Introduction

Depression and obesity are two of today’s major public health issues that cause a magnitude of disease burden, functional disability and mortality [1–7]. According to the World Health Organization (WHO), ~350 million people worldwide suffer from depression [6], and 600 million people are obese [7].

Obesity is an excessive fat accumulation that may impair health [7], and is defined by the WHO as having a body mass index (BMI) ≥30 kg/m^2^ [7]. It has been reported to increase the likelihood of various diseases, particularly heart disease, type 2 diabetes, obstructive sleep apnoea, certain types of cancer, and osteoarthritis [8]. It most commonly develops on the basis of interactive factors: a combination of excessive food energy intake against the background of the modern obesogenic food environment, the lack of physical activity (PA) in contemporary daily life, and genetic susceptibility [8–10]. The modern food environment is characterized by readily available snacks, caloric beverages, foods with high palatability and high energy density, large portion sizes, comparatively low price, and ready availability [9]. Of the main causal factors, caloric intake and PA remain best suited to therapeutic influence.

Physical activity is inversely related to body weight and fat mass as shown, for example, in the European prospective investigation into cancer and nutrition (EPIC) study [10] using data from more than 4,00,000 study participants; for further review on studies regarding the relationship between PA, fat mass and obesity, see [11, 12]. Neuropeptides and cytokines seem to modify this association as plasma levels of the neuropeptide orexin-A have been shown to be lower in patients with obesity, but higher in participants with obesity who are more physically active [13], and levels of interleukin (IL-4), IL-10 and IL-13 have been reported to be elevated in participants with low PA [14].

Medical literature clearly demonstrates beneficial effects of PA on several health outcomes, including its role as a protective factor against cardiovascular disease and all-cause mortality [15]. Regular PA has been shown to reduce symptoms of anxiety and depression and to increase both physical and psychological quality of life [16, 17]. Worldwide, however, one out of every five adults is considered physically inactive, which is defined as being engaged in <20 min per day of vigorous-intensity PA on at least 3 days per week or <30 min per day of moderate PA on at least 5 days a week [18, 19]. The most objective measure to determine PA is actigraphy [20, 21] which makes a record of the activity level of the body. These actigraphy devices use various technologies such as piezoelectric effects to measure acceleration along a movement axis [22]. Typically, the device is worn for a specific period of time to continuously record gross motor activity using an accellerometer. Actigraphy has been in use since the 1980s to quantify PA [23].

A reciprocal relationship between obesity and depression has been observed. Obesity has been reported to increase the risk for depression [24–30] and, in turn, depression has been shown to increase the risk for obesity [31–38] in prospective studies. Typical symptoms of depression include fatigue and loss of energy [39]. Individuals with such symptoms are more likely to engage in lower levels of PA, which, in turn, facilitates the development of obesity. Indeed, depressive symptoms have been found to be associated with very little or even no PA [40–42], and case control studies showed less PA in acutely depressed patients compared to remitted patients or healthy controls [43–47]. Moreover, scientific data suggest that PA is a preventative measure against depressive symptoms in patients with obesity [42]. This finding indicates that research around obesity, depression and PA might be of clinical relevance for patients with obesity to avoid the development of depression as co-morbid with obesity.

However, we are not aware of any study that has specifically investigated the level of PA in lean depressed patients compared to lean healthy controls. Similarly, we did not find any investigation examining the difference between depressed and non-depressed patients with obesity. It is yet unclear whether patients suffering from both obesity and depression are less physically active than patients with obesity but without depression. This question bears a significant clinical impact, because if patients with both obesity and depression are less physically active than non-depressed individuals with obesity, it would put them at an additional risk for sequelae of obesity. As depression is a treatable disorder, this additional risk may be avoidable. Evaluation of the additional risk of low PA due to depression for patients suffering from another disease is not a novel research idea. The risk of low PA due to depression has, for example, also been evaluated for patients suffering from chronic obstructive pulmonary disease (COPD) [48] and fibromyalgia [44]. There are some published studies using actigraphy to determine differences in PA between patients with and without depression [44] (for review see [47]), and also between patients with and without obesity [10, 11]. Yet again, to the knowledge of the authors, studies comparing the actigraphy-measured activity of depressed obese patients and non-depressed patients are not available. If depressed patients with obesity were less physically active than non-depressed patients with obesity, the group of patients suffering from both obesity and depression would be at a specifically high risk for health consequences of inactivity as well as for reduced wellbeing and limited quality of life. For the measurement of activity in the present study, we used a SenseWear™ Armband (SWA) which is an established actometric method [49–51] to objectively measure PA.

Taken together, no studies are available comparing patients with both depression and obesity and patients with obesity but not depression with regard to objectively measured PA. The present study first investigates whether patients with obesity are less physically active than healthy controls and, secondly, whether patients who are both depressed and obese are less physically active than non-depressed patients with obesity.

## Methods

### Participants

In total, we recruited 304 participants from the outpatient clinic of the Integrated Research and Treatment Center for Adiposity Diseases Leipzig (IFB), from the Department of Psychiatry and Psychotherapy of the University Hospital Leipzig and via announcements (intranet, internet, local newspapers). All participants were aged between 18 and 70 years.

For the present study, we selected from the pool of available participants who had partaken in an actigraphy recording and whose actigraphy data fulfilled certain quality criteria (see below). According to their BMI and their BDI2 total score, those participants (*N* = 188) were classified as belonging to three groups: (a) *N* = 47 patients with depression (BDI2 score >13 points) and obesity (BMI ≥30); (b) *N* = 70 non-depressed (BDI2 score ≤13 points) patients with obesity; and (c) *N* = 71 non-depressed and non-obese (BMI < 30) control participants.

Socio-demographic data are shown in Table 1. There was no significant difference in gender ratio (*χ* ^2^ = 0.755, *p* = ·686) between the three groups. However, a one-way ANOVA revealed a significant difference in mean age [healthy controls: 34.3 (±12.0) years, non-depressed patients with obesity: 43.3 (±13.2) years, patients with obesity and depression: 43.6 (±12.5) years; *F*_(2,185)_ = 11.553, *p* < .001], since the control group was significantly (*p* < .001) younger compared to the two obese groups, who did not differ in mean age. Although not statistically significant, the number of smokers was highest in the obese-depressed group compared to the other two groups yet, as mentioned, this difference did not reach significance (*χ* ^2^ = 2.399, *p* = .301). For obvious reasons, the three groups differed significantly concerning BMI [healthy controls: 23.4 (±3.3), obese/non-depressed: 43.7 (±7.4), obese/depressed: 47.0 (±8.1); *F* = 258.094, *p* < .001] and BDI2-scores (*F*_(2,185)_ = 182.655, *p* < .001), as the controls had lower BMI scores compared to the two obese-groups (*p* < .001), whereas the obese-depressed groups scored higher in the BDI2 compared to the two non-depressed groups (*p* < .001).

**Table 1:** Sample characteristics

	Healthy controls	Non-depressed patients with obesity	Patients with both obesity and depression	
*N*	71	70	47	
Gender [f/m]	48/23	51/19	31/16	*χ* ^2^ = 0.755, *p* = .686
Age (mean ± SD)	34.32 ± 12.041	43.33 ± 13.225	43.62 ± 12.538	*F* = 11.553, *p* < .001
Smoker [yes/no]	13/58	14/56	33/14	*χ* ^2^ = 2.399, *p* = .301
BMI (mean ± SD)	23.36 ± 3.299	43.73 ± 7.412	47.04 ± 8.098	*F* = 258.094, *p* < .001
BDI2 score (mean ± SD)	4.27 ± 4.188	5.54 ± 3.929	21.89 ± 7.927	*F* = 182.655, *p* < .001

*BMI* body mass index, *BDI2* beck depression inventory, second edition

### Measures and procedure

For recruitment, potential participants were contacted via phone and invited to participate in a telephone screening interview, comprising socio-demographic data, screening for somatic disorders and a checklist of the structured clinical interview for DSM-IV [52]. Eligible participants were then invited to the study center, where exclusion criteria were assessed in more detail. Exclusion criteria were acute or chronic infections, current medication with a recognized major impact on the immune system, current psychiatric medication, psychiatric and neurological disorders apart from depression, and a history of head injury with loss of consciousness exceeding 1 h. Assessments for current and past history of physical and mental health problems as well as current medication were performed using standardized forms.

Study participants underwent a full physical examination by a study physician. Qualified healthcare professionals performed all examinations. Weight [kg] was determined in underwear and without shoes using a digital scale calibrated and standardized using a weight of known mass. Height [cm] was recorded using a stadiometer with participants standing on a flat surface at a right angle to the vertical board of the stadiometer. BMI [kg/m^2^] was defined as body weight [kg] divided by the square of height [m^2^].

After inclusion in the study, participants answered questionnaires including the German version of the revised Beck Depression Inventory second edition, (BDI2) [53, 54]. The German version of the BDI2 demonstrated good reliability and validity in clinical and nonclinical samples for assessing self-rated severity of depression and for assessing the course of depressed symptoms under treatment [55]. We used the cutoff score of 13 in accordance with Beck’s original manual for evaluation of the BDI2 [55]. This cutoff was also applied in previous studies using the German version of the BDI2, where depressed inpatients showed a mean BDI2 score around 33, whereas non-clinical samples exhibited a mean BDI2 score of <8 [54].

In a subgroup of the original sample, a 1-week actigraphy recording was performed, using the SenseWear^®^ Pro 3 actigraph (SWA; BodyMedia Inc.; Pittsburgh, Pennsylvania). This device is attached to the upper right arm and records 2-axis body acceleration, skin temperature, heat flux and galvanic skin response. Furthermore, the device detects periods in which it is not worn (off-arm periods). Recordings were collected between the telephone screening and assessment day. Participants kept a sleep and activity diary throughout the recording period. Actigraphic data were analyzed using SenseWear^®^ Professional Software Version 7 (BodyMedia Inc.) for estimation of number of steps and METs per 1-min timeframe using validated proprietary scoring algorithms included in the software. A MET is a physiological measure expressing the energy cost of PA.

Several studies have demonstrated that the SWA device provides accurate estimates of energy expenditure during rest and daily life activities, comparable to the gold standards of indirect calorimetry and doubly labeled water [49–51, 56–59].

Score data were entered into a customized Excel-Template for further data preparation. According to sleep scoring from the software and sleep logs provided by the participants, the wake phase (i.e., daytime) was determined as between two consecutive night sleep intervals. For each daytime interval, the variables’ steps (=sum of steps per 1 min-segment) and METs (=mean of all MET values per 1 min-segment) were calculated and afterwards averaged to obtain mean values for the total week. Datasets were included in the analysis if they contained analyzable data for at least 5 daytime intervals, i.e., at least 3 week days and both weekend days.

## Results

### Steps

**Fig. 1:**
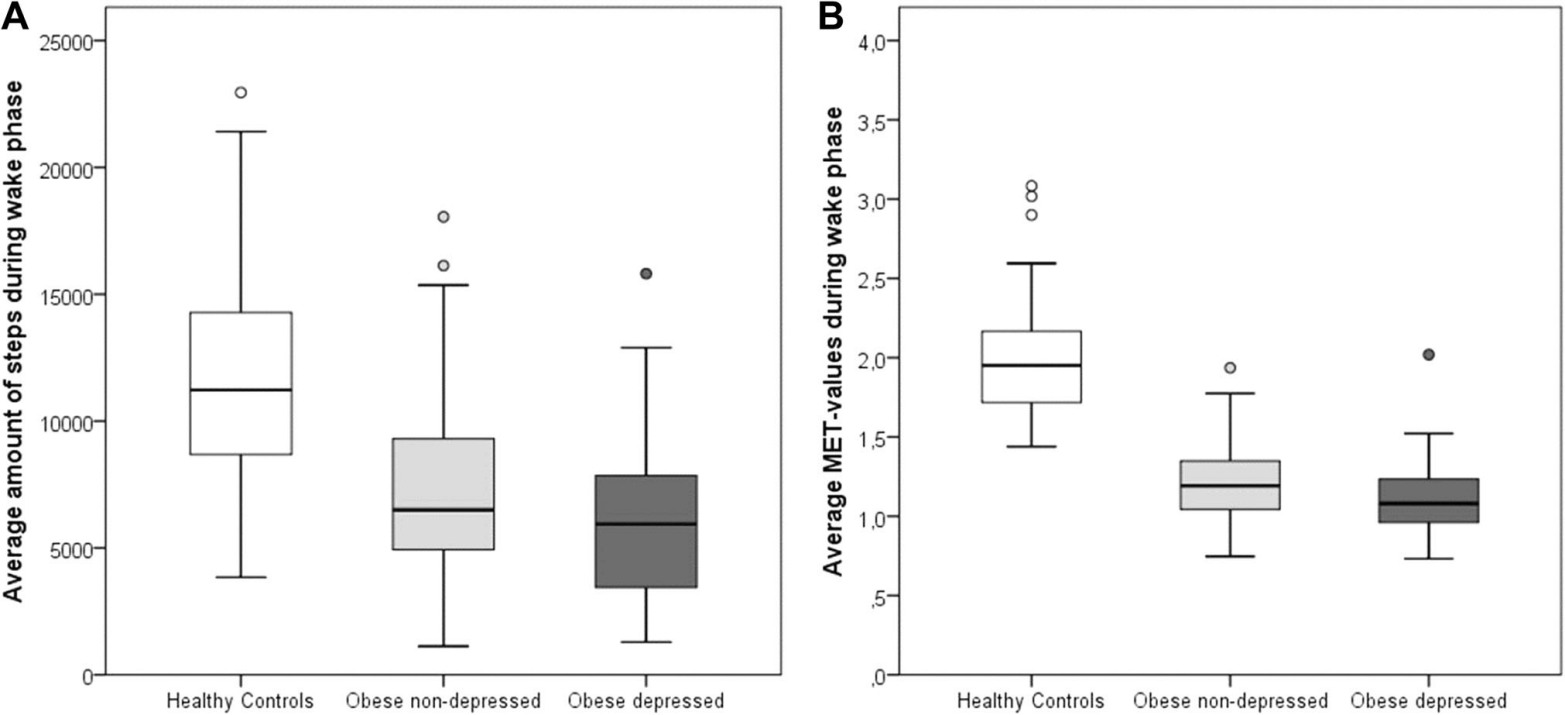
Amount of steps (part **a** *left*) and MET values (part **b** *right*) of healthy controls, non-depressed patients with obesity and patients with both depression and obesity

The average amount of steps taken per day differed among the three samples [healthy controls: 11,585.6 (Mean) ± 3730.68 (SD); obese/non-depressed: 7282.9 ± 3546.60; obese/depressed: 6177.0 ± 3291.19; see also Fig. 1a]. Since there was a significant correlation between steps and age in the total sample (steps: 8631.4 ± 4245.48; age: 40.0 ± 13.31 years; *ρ* = −0.265, *p* < .001, we included age as a covariate and performed an ANCOVA, which revealed a significant main effect of group (*F*_(2,184)_ = 33.665; *p* < .001: *η* ^2^ = 0.268) but not of age (*F*_(1,184_) = 0.912; *p* = .341; *η* ^2^ = 0.005). Post hoc comparisons of the three groups proved that healthy controls differed from obese/non-depressed (*p* < .001) as well as obese/depressed (*p* < .001) participants, whereas the latter two did not differ from each other (*p* = .103).

**Fig. 2:**
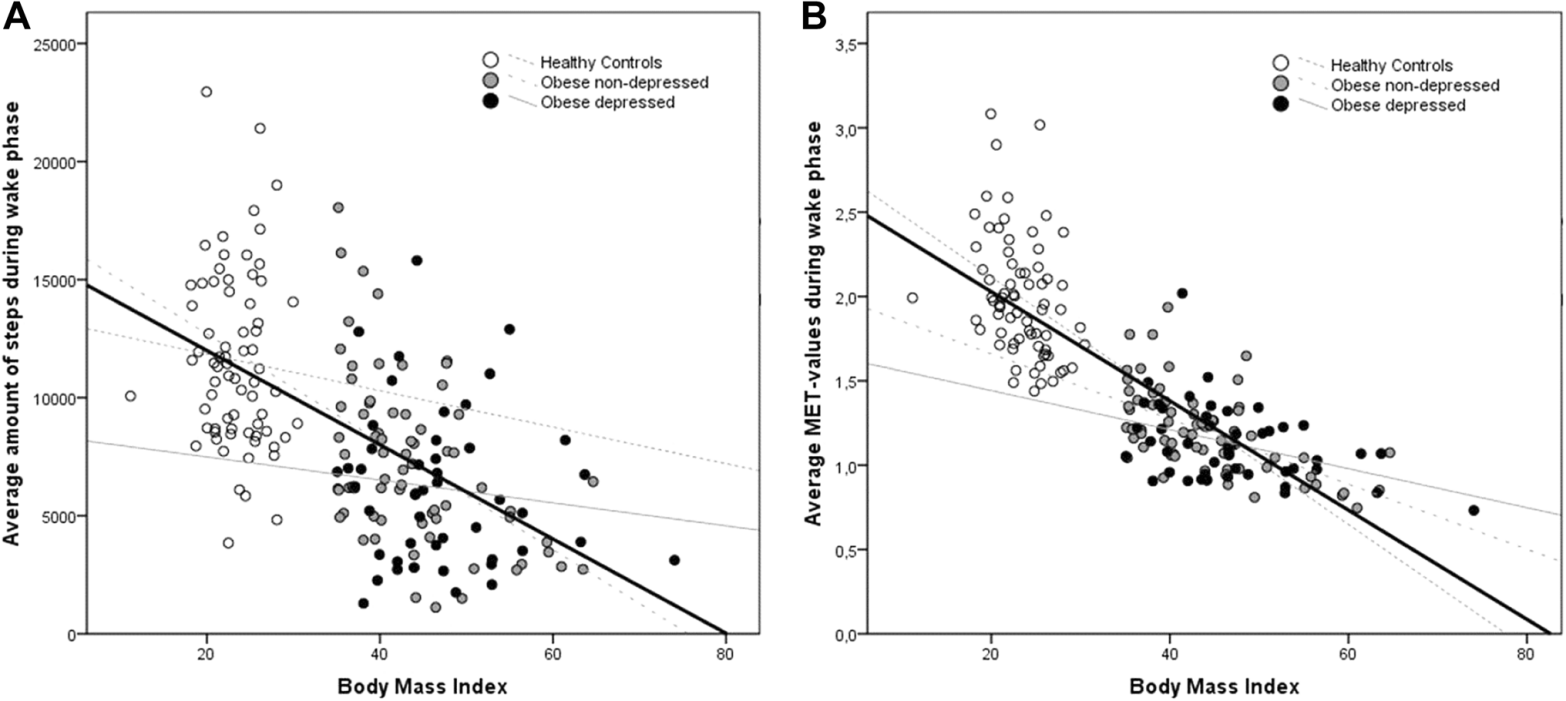
Relationship between BMI [kg/m^2^] and the amount of steps walked during the wake phase (**a** *left*) and between the BMI and the MET values during the wake phase (**b** *right*) of healthy controls, non-depressed patients with obesity and patients with both depression and obesity. The *bold black* *line* depicts the regression line for the total group. The correlations were significant in the total group as well as in the subgroup of patients with obesity but without depression

In the total sample, there was a significant negative correlation between BMI and average amount of steps (*ρ* = −0.605, *p* < .001; see also Fig. 2a), which was only seen in the obese/non-depressed subgroup (*ρ* = −0.450, *p* < .001), but not in healthy controls (*ρ* = −0.105, *p* = .384) or the obese/depressed subgroup (*ρ* = −0.100; *p* = .503).

### METs

The three groups also had different average MET values per day [healthy controls: 1.99 (Mean) ± 0.361 (SD); obese/non-depressed: 1.20 ± 0.247; obese/depressed: 1.13 ± 0.228; see also Fig. 1b]. Again, there was a significant correlation between age and METs in the total sample (METs: 1.48 ± 0.494; age: 40.0 ± 13.31 years; *ρ* = −0.308, *p* < .001). Thus, age was again included as covariate into the ANCOVA, which revealed a significant main effect of group (*F*_(2,184)_ = 152.627; *p* < .001; *η* ^2^ = 0.624) but not of age (*F*_(1,184)_ = 0.181; *p* = .671; *η* ^2^ = 0.001). Post hoc comparisons of the three groups again resulted in significant differences between healthy controls and obese/non-depressed (*p* < .001) as well as obese/depressed (*p* < .001) participants, but no significant differences were observed between the two obese groups (*p* = .189).

There was a significant and strong negative correlation between BMI and average MET values (*ρ* = −0.836, *p* < .001; see also Fig. 2a) in the total sample. A moderate negative but significant association was also seen in the obese/non-depressed subgroup (*ρ* = −0.587; *p* < .001), whereas only weak associations were seen in healthy controls (*ρ* = −0.415, *p* < .001) and the obese/depressed subgroup (*ρ* = −0.363, *p* = .012).

## Conclusions

In the present study, we found significantly less PA in patients with obesity compared to healthy controls, which aligns with previous studies finding lower levels of activity in obese patients [10, 11]. According to our findings using an SWA, healthy controls walked a mean of roughly 11,500 steps per day, patients with obesity but not depression walked approximately 7000 and patients with both obesity and depression walked about 6000 steps per day. We also used METs as a measure of PA. Regarding this physiological measure reflecting the energy cost of PA, the lower level of PA associated with obesity was likewise substantial, whereas an additional depressive symptomatology was not associated with a further significantly lower energy expenditure. Moreover, we found a significant negative association between PA and the BMI as a continuous variable for the total group and the subgroup of non-depressed patients with obesity.

Results of the previous studies vary considerably, as they were performed in different countries, span different age groups and use different definitions of healthy weight range in addition to varying instruments to measure the steps walked per day or METs per day [60–63]. However, independent of the cultural background and the age range, they found differences between patients with obesity and individuals with a healthy weight. Overall, their walked steps data are similar to the range we found in our investigation [60–63].

For example, a French study regarding PA in adolescents with obesity found that individuals with a healthy weight walked about 10,000 steps per day, whereas patients with obesity did about 9000 as measured by an SWA [60]. In this study, the METs were analyzed revealing a value of 1.8 for healthy weight and 1.3 for study participants with obesity which is quite close to the MET values we obtained (see Fig. 1b). A North American investigation using pedometers found a mean of about 6000 steps per day in healthy weight adults and about 4000 steps in participants with obesity [61]. In a British study of older adults, normal weight participants were significantly more active (with a mean of more than 5000 steps per day) than patients with obesity (mean about 3000 steps per day) [62]. In a Czech study using the waist-hip-ratio (WHR) as an indicator for excess weight and obesity, healthy study participants walked about 8000 steps as measured by pedometry, whereas participants with a risky WHR walked about 7000 steps [63].

As previous studies also showed reduced PA in depressed patients [43–47], our aim was to investigate whether participants with both obesity and depression would be even less physically active than patients with obesity but without depression. Although our data show a trend in this direction, the results were not statistically significant. These results demonstrate that obesity alone is associated with an almost 50% reduction of walked steps per day. Given an average stride length of about 73 cm, this would mean that healthy controls in our study allegedly walked about 8 km, whereas patients with obesity but without depression would walk approximately 5 km per day and patients with both obesity and depression would walk about 4.5 km. When interpreting our data, one should also consider that obesity and depression are closely related [24–38] which means that it is difficult to disentangle and isolate the effects of obesity and the effects of depression, because there may be common causes underlying both obesity and depression [64].

If we had found a significant difference in PA levels of patients with obesity and depression in contrast to non-depressed patients with obesity, this could have provided robust support for recommending a depression screening for every obese patient who needed an increase in PA. But as there is only a trend in this direction, treatment against depression does not seem to be of major relevance for increasing PA in the subgroup of obese and depressed patients. However, depression in itself is a disorder which merits treatment [65], and which might have a huge impact on the quality of life of obese patients [66]. Therefore, one has to keep in mind that reduced PA is only one single clinical feature of obese patients among others which might be related to depression. Our study would have profited from a second control group of patients with depression but not obesity. This would have helped to separately evaluate the influence of obesity and depression. Future studies should consider such a methodological improvement.

There are several further shortcomings of our study, the first of which is the sample size. One could argue that if we had a larger study with more study participants, the effect of depression in patients with obesity would most likely have become significant. This expected increased likelihood of significant results indicates that the sample size used in additional studies on this topic should be larger. One should consider that in clinical terms, it may of course be relevant for a specific obese patient to receive treatment for depression, because the additional steps a patient would undertake after a successful treatment of depression might keep the patient under the threshold for diabetes [67] or other adverse health consequences of obesity.

As it is known from previous studies that PA declines with age [68, 69], another shortcoming of our study was that the group of healthy controls was younger than both groups of obese patients. In addition, we found a significant correlation between PA and age in our sample which underlines how our data are representative and may, therefore, be considered reliable. As reported in “Results”, we thus included age as a covariate when calculating comparisons in terms of PA between the groups. However, it would have been methodologically desirable to have comparable age groups when comparing healthy and obese participants.

Furthermore, patients taking medication which would influence appetite and weight were not excluded from the study, because we wished to minimize the loss of recruited obese patients. Therefore, both groups of obese patients include some patients taking antidiabetics such as metformin, which decreases appetite and can lead to weight loss [70]. Thus, we have to bear in mind that this study is based on a naturalistic sample of obese patients and healthy controls.

A strength of this study, however, is the use of the SWA to measure PA objectively. Although we can report a negative association between obesity and PA, we are not able to determine the direction of causality. PA may lead to weight loss, but higher body weight may also lead to a lower PA level. This converse causality may be the reason why some interventions with increased PA do not have a statistically significant effect on body weight [71].

However, even if a patient with obesity does not lose weight by increasing their PA, the PA might help to mitigate negative consequences of obesity like the inflammatory changes in the body or the development of depression [14, 42]. For example, we could show that increased PA in obese patients is associated with a decrease in cytokine production, which most likely reflects a reduction in obesity-induced inflammatory processes [14], and Dankel et al. could show that PA might be a preventative measure against depressive symptoms in patients with obesity [42]. Therefore, patients suffering from obesity should be encouraged to be physically active.

Taken together, we investigated PA in healthy controls and patients of a categorically obese BMI with or without depression using an SWA by comparing METs spent and steps walked. We found that obesity was associated with a substantial reduction of PA and energy expenditure, whereas the effect of depressive symptoms was comparably small.
